# The Role of Ubiquitination and Deubiquitination in the Pathogenesis of Acute Kidney Injury: Progress in Research

**DOI:** 10.3390/biomedicines13122873

**Published:** 2025-11-25

**Authors:** Yu Zhou, Shuchun Tao, Lei Liu, Lei Zhang

**Affiliations:** 1 Institute of Urology, Surgical Research Center, School of Medicine, Southeast University, Nanjing 210009, China; 18051988005@163.com (Y.Z.); 17755357445@163.com (S.T.); 2 Department of Urology, Zhongda Hospital, Southeast University, Nanjing 210009, China; 3 Department of Pathology, Affiliated Hospital of Nantong University, Nantong 226001, China

**Keywords:** acute kidney injury, ubiquitin-proteasome system, ubiquitin-like modifiers, deubiquitinating enzymes, targeted therapy

## Abstract

Acute kidney injury (AKI) remains a critical clinical challenge due to its complex pathophysiology and lack of targeted therapies. We hypothesize that the ubiquitin-proteasome system (UPS) and ubiquitin-like modifiers (UBLs) are not merely isolated players but constitute an intricate and coordinated regulatory network whose dysregulation is a central driving force in AKI progression. This review synthesizes the pivotal roles of the ubiquitin–proteasome system and ubiquitin-like modifiers in driving AKI progression, emphasizing their regulation of inflammatory responses, cell death pathways (apoptosis, pyroptosis, ferroptosis), mitochondrial dysfunction, and aberrant repair. We detail enzymatic cascades involving E1-E2-E3 ligases, deubiquitinating enzymes, and ubiquitin-like modifiers (SUMOylation, neddylation, ISGylation, UFMylation), highlighting their integration into a coordinated network modulating NF-κB/NLRP3 signaling, mitophagy, and growth factor pathways, thereby providing novel strategies for targeted therapy.

## 1. Introduction

Acute kidney injury (AKI) is a common clinical renal disorder, and its occurrence is closely associated with various pathological mechanisms, including oxidative stress, inflammatory response, apoptosis, endoplasmic reticulum stress, and mitochondrial damage [1]. The onset of AKI not only significantly impairs renal function but is also often associated with multi-organ failure. This is due to the accumulation of metabolic products and toxins in the body following renal injury, which subsequently affects the function of other organs [2]. Furthermore, renal injury triggers the release of inflammatory mediators, leading to systemic inflammatory response syndrome (SIRS), which exacerbates multi-organ dysfunction [3]. The mortality rate in AKI patients is relatively high, primarily due to various adverse outcomes associated with AKI, such as multi-organ dysfunction, electrolyte disturbances, and acid–base imbalance [4,5]. AKI patients often have comorbid conditions such as cardiovascular diseases, diabetes, and hypertension, which further increase their mortality risk [6,7]. However, the underlying molecular mechanisms of AKI remain unclear, which has hampered the development of preventive and therapeutic strategies. Therefore, further investigation into the specific molecular mechanisms of AKI pathogenesis is essential [8].

Ubiquitination is a widespread cellular regulatory process that involves the attachment of one or more ubiquitin molecules to target proteins, thereby regulating their function, stability, localization, and degradation [9]. Ubiquitination plays a crucial role in many important biological processes in cells, including protein degradation, signal transduction, cell cycle regulation, and responses to external stressors [10]. Dysregulation of ubiquitination is closely linked to the development of various diseases, particularly in the occurrence and progression of AKI [11]. Ubiquitination not only regulates the homeostasis of renal cells but also plays a role in the repair process after kidney injury, affecting biological processes such as inflammation, apoptosis, and mitochondrial function [12].

In addition to classical ubiquitination, several ubiquitin-like modifications (UBLs) have been identified as important parallel regulatory systems in cells, participating in a wide range of physiological and pathological processes. These include small ubiquitin-like modifier (SUMO) conjugation (SUMOylation), Neural Precursor Cell-Expressed Developmentally Downregulated 8 (NEDD8) conjugation (Neddylation), Interferon-Stimulated Gene 15 (ISG15) conjugation (ISGylation), Ubiquitin-Fold M odifier 1 (UFM1) conjugation (UFMylation), HLA-F-Adjacent Transcript 10 (FAT10) conjugation (FATylation), among others [13]. These modifications fine-tune intracellular signaling pathways and cellular homeostasis by modulating protein function, stability, and interactions [14]. Increasing evidence indicates that these ubiquitin-like modifications act in concert with classical ubiquitination to regulate cellular stress responses, inflammation, and repair in AKI [15].

Therefore, rather than viewing these pathways as isolated processes, we interpret them as components of a coordinated ubiquitin-related regulatory network that dynamically determines renal cell fate under stress. Based on this concept, this review systematically summarizes the roles of ubiquitination, deubiquitination, and multiple UBLs in AKI pathogenesis, highlighting their mechanistic interactions and therapeutic potential.

In preparing this review, we comprehensively examined studies published up to September 2025 in PubMed, Web of Science, and Scopus databases, focusing on mechanistic and therapeutic insights into ubiquitination, deubiquitination, and ubiquitin-like modifications in AKI.

## 2. Overview of Ubiquitination and Ubiquitin-like Modifications

### 2.1. Mechanism of Ubiquitination

**Figure 1:**
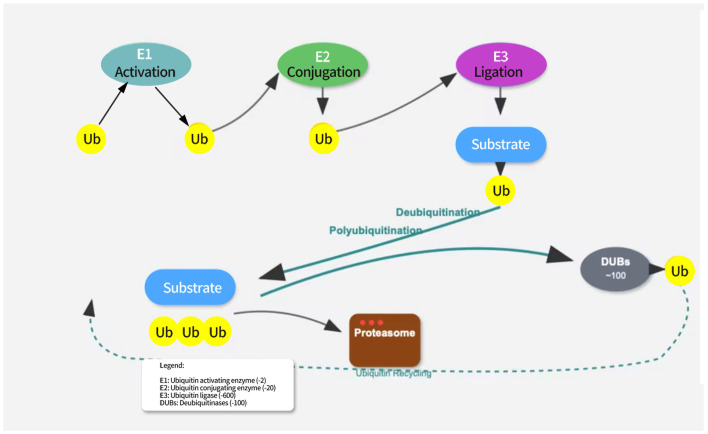
The ubiquitination pathway involves three sequential enzymatic steps. E1 (ubiquitin-activating enzyme) activates ubiquitin in an ATP-dependent manner. E2 (ubiquitin-conjugating enzyme) receives the activated ubiquitin from E1. E3 (ubiquitin ligase) recognizes the target substrate and facilitates the transfer of ubiquitin from E2 to the substrate. Polyubiquitinated proteins are targeted for proteasomal degradation. Deubiquitination is mediated by deubiquitinating enzymes (DUBs) that remove ubiquitin from substrates, allowing for ubiquitin recycling.

Ubiquitination is an important post-translational modification of proteins, primarily mediated through the E1-E2-E3 enzyme cascade system. The E1 enzyme (ubiquitin-activating enzyme) first activates the ubiquitin molecule, linking it to its own cysteine residue via a thioester bond. The activated ubiquitin is then transferred to the E2 enzyme (ubiquitin-conjugating enzyme), which, in collaboration with the E3 enzyme (ubiquitin ligase), attaches the ubiquitin molecule to the substrate protein [16]. The E3 enzyme plays a critical role in substrate specificity recognition, determining which proteins will undergo ubiquitination [17]. Different E3 enzymes can recognize distinct substrate proteins, enabling the specific modification of proteins (see Figure 1).

The eight ubiquitin attachment sites (M1, K6, K11, K27, K29, K33, K48, K63) can each form complex ubiquitin chains by linking to the C-terminus of another ubiquitin molecule [18]. Ubiquitin chains linked by K48 are primarily associated with protein degradation [19]. When a protein is modified by a K48-linked ubiquitin chain, it is recognized by the proteasome and subsequently degraded. In addition, other types of ubiquitin chains, such as K11/K48 hybrid chains, play a critical role in the cell cycle and protein quality control [20,21,22]. Engineered bispecific antibodies have been used to detect K11/K48-linked ubiquitin chains, and mitotic regulators, misfolded nascent peptides, and pathogenic Huntington protein variants were identified as endogenous substrates. These ubiquitin chains promote the rapid proteasomal clearance of aggregation-prone proteins [23,24].

### 2.2. Deubiquitination

**Figure 2:**
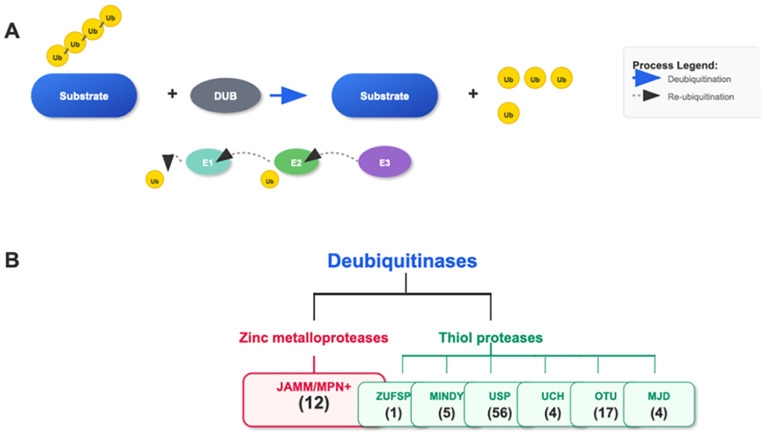
Ubiquitination/Deubiquitination and the Cascade of the DUB Family. (**A**) The E1, E2, and E3 cascade adds single or polyubiquitin chains to the substrate. DUBs remove the ubiquitin attached to the substrate. (**B**) The DUB family consists of seven subgroups: JAMM/MPN+, ZUSP, MINDY, USP, UCH, OTU, and MJD, classified based on the characteristics of their conserved domains. The number of genes in each family is indicated.

Deubiquitinating enzymes (DUBs) form an important family within the ubiquitin system. This family mainly includes ubiquitin-specific proteases (USP), otubain (OTU), ubiquitin carboxyl-terminal hydrolases (UCH), and JAB1/MPN/Mov34 metalloenzymes (JAMM) families [25,26]. These DUBs have distinct structural and functional features that enable them to specifically recognize and cleave the linkage between ubiquitin and substrate proteins, thereby removing the ubiquitin modifications from proteins [27,28] (see Figure 2).

DUBs play a crucial role in maintaining protein homeostasis by precisely regulating the ubiquitination state of proteins [29,30,31]. Its regulatory mechanism is mainly reflected in the following aspects:

Steady state regulation in DNA damage repair: In Fanconi anemia research, it was found that the deubiquitinase USP1 can remove monoubiquitination modifications from FANCD2, allowing the cell cycle that has been stalled due to damage repair to continue, thereby maintaining normal cell function [32,33]. Similarly, Guo X et al. [34] showed that USP4 is the ubiquitination enzyme of BRCA1, which positively regulates the stability and function of BRCA1 through ubiquitination, and plays a key role in the inhibition of breast cancer. Robert Hromas et al. [35] further found that in tumor cells with BRCA1 mutations, increased genomic instability leads to the accumulation of unfolded proteins. BRCA1 acts as an E3 ligase in the endoplasmic reticulum, ubiquitinating the unfolded protein reaction (UPR) sensors PERK and IRE1 and subsequently degrading them via the proteasome. When BRCA1 is mutated or deleted, the levels of PERK and IRE1 proteins increase, leading to sustained activation of UPR, inhibition of protein folding, or UPR signaling significantly reduces the overall survival rate of BRCA1-deficient cancer cells [36].

Regulation of signal transduction pathways: DUBs can affect the precise regulation of intracellular signaling pathways. Research has shown that ubiquitination regulates key factors involved in abscisic acid (ABA) synthesis and signal transduction, affecting plant response to ABA and participating in plant growth, development, and stress response processes. During this process, deubiquitinase may affect signal transduction by regulating ubiquitination levels [37].

Cell protection and stress response: DUBs help cells cope with various stresses and injuries. Yihao Liao et al. [38] demonstrated that OTU domain ubiquitin aldehyde binding protein 1 (OTUB1), as the most important member of the OTU DUB superfamily, is a key regulatory factor in various physiological processes, including DNA damage repair [39]. DUBs regulate the stability and function of proteins involved in DNA repair by removing ubiquitin, thereby promoting DNA damage repair [40]. Research has found that saffron cultured cells provide protective effects on peripheral blood cells of irradiated mice, which may be related to the role of deubiquitinase in cell protection [41].

In summary, DUBs maintain cellular protein homeostasis through multi-level regulatory mechanisms, and their dysfunction often leads to disease occurrence, providing an important perspective for understanding the complexity of protein homeostasis regulation (Table 1).

### 2.3. Ubiquitin-like Modifications, UBLs

Ubiquitin-like modifications represent a diverse family of post-translational modifications that share structural or functional similarities with ubiquitination. While they utilize similar enzymatic cascades for protein conjugation, they involve distinct modifiers and serve unique cellular functions. Here, we discuss the major UBLs and their roles in cellular processes relevant to AKI (Table 2).

#### 2.3.1. SUMOylation

SUMOylation is a post-translational modification involving the covalent attachment of small ubiquitin-like modifier (SUMO) proteins to target substrates. The SUMO family in mammals consists of four isoforms: SUMO-1, SUMO-2, SUMO-3, and SUMO-4. SUMO-2 and SUMO-3 share 97% sequence identity and are often referred to as SUMO-2/3, while SUMO-1 shares only about 50% sequence identity with SUMO-2/3 [42].

The SUMOylation process involves a three-step enzymatic cascade similar to ubiquitination: activation by the E1 enzyme (SAE1/SAE2 heterodimer), conjugation by the E2 enzyme (UBC9), and ligation by E3 ligases (e.g., PIAS family proteins, RanBP2, and Pc2). SUMOylation typically occurs at lysine residues within the consensus sequence ΨKxE (where Ψ is a hydrophobic amino acid, K is the target lysine, x is any amino acid, and E is glutamic acid). However, non-consensus SUMOylation has also been reported [43].

In the context of cellular stress, SUMOylation is particularly important for maintaining genomic stability and mediating stress responses. Research by Chatzikalil E [44] has shown that SUMO E3 ligases are involved in regulating protein stability and function, highlighting the importance of SUMOylation in cellular processes, including stress responses that are relevant to AKI pathophysiology.

#### 2.3.2. Neddylation

Neddylation is a ubiquitin-like modification involving the conjugation of Neural precursor cell expressed developmentally downregulated protein 8 (NEDD8) to target proteins. Similarly to ubiquitination, neddylation proceeds through a cascade of E1 (NEDD8-activating enzyme, NAE), E2 (UBC12/UBE2M or UBE2F), and E3 enzymes (RBX1/2, DCNL1-5) [45].

The most well-characterized substrates of neddylation are the cullin family proteins, which are components of Cullin-RING E3 ubiquitin ligases (CRLs). Neddylation of cullins activates CRLs by inducing conformational changes that bring the E2-ubiquitin complex closer to the substrate, thereby enhancing ubiquitin transfer. Through this mechanism, neddylation indirectly regulates the ubiquitination and subsequent degradation of numerous proteins involved in cell cycle progression, signal transduction, and stress responses [46].

Beyond cullins, other neddylation substrates include p53, Murine double minute 2 (MDM2), ribosomal proteins, and histones, indicating a broader role of neddylation in cellular regulation [21].

In the context of kidney function, neddylation has been implicated in: (1) Cell cycle regulation: By activating CRLs, neddylation promotes the degradation of cell cycle inhibitors, facilitating cell proliferation. (2) DNA damage response: Neddylation of DNA damage response proteins modulates their activity and stability. (3) Oxidative stress: NEDD8 has been shown to inhibit PARP-1 activation, potentially reducing oxidative stress-induced cell death [47]. (4) Protein quality control: By regulating CRL activity, neddylation influences the degradation of misfolded or damaged proteins.

Importantly, the neddylation inhibitor pevonedistat (MLN4924) has shown promising results in preclinical models of kidney injury, suggesting that targeting neddylation may be a viable therapeutic approach for AKI [48].

#### 2.3.3. ISGylation

ISGylation refers to the conjugation of interferon-stimulated gene 15 (ISG15) protein to target substrates. ISG15 is strongly induced by type I interferons, viral infections, and bacterial lipopolysaccharides, indicating its crucial role in immune responses [54,55].

The ISGylation process involves an enzymatic cascade consisting of an E1 enzyme (UbE1L), an E2 enzyme (UbcH8), and E3 ligases (primarily HERC5 and TRIM25). ISGylation can be reversed by the ISG15-specific protease USP18, which also functions as a negative regulator of type I interferon signaling.

ISG15 functions through two distinct mechanisms:

Conjugation to target proteins (ISGylation): Modifies the function, stability, or interactions of target proteins [39].

Non-conjugated (free) ISG15: Acts as a cytokine-like molecule that can be secreted and bind to cell surface receptors [37].

Given its roles in immune modulation and stress responses, ISGylation represents a potential therapeutic target for inflammatory and oxidative stress-related aspects of AKI [49].

#### 2.3.4. UFMylation

UFMylation is a more recently discovered ubiquitin-like modification involving the conjugation of Ubiquitin-fold modifier 1 (UFM1) to target proteins. The UFMylation cascade consists of an E1 enzyme (UBA5), an E2 enzyme (UFC1), and an E3 ligase (UFL1). The process can be reversed by the UFM1-specific protease UFSP2 [50].

UFM1 is expressed in various tissues, with particularly high levels in the liver, pancreas, and heart. In the context of cellular function, UFMylation plays crucial roles in: (1) Endoplasmic Reticulum (ER) function: UFMylation regulates ER homeostasis and the UPR, which are critical for cell survival under stress conditions. (2) Protein quality control: The UFM1 system contributes to the recognition and processing of misfolded proteins. (3) Autophagy regulation: UFMylation influences autophagy pathways, affecting intracellular material metabolism and clearance mechanisms. (4) Cell cycle progression: UFM1 modification regulates proteins involved in cell cycle control [56,57].

#### 2.3.5. FATylation

FATylation refers to the conjugation of FAT10 (HLA-F-adjacent transcript 10, also known as ubiquitin D) to substrate proteins, and its expression is primarily induced by inflammatory cytokines such as TNF-α and IFN-γ [51]. The FATylation process relies on the E1 enzyme UBA6 and the E2 enzyme USE1, while the specific E3 ligases remain incompletely characterized [52].

In a model of AKI progression to chronic kidney disease (CKD), FAT10 modifies β-catenin in renal tubular epithelial cells through FATylation, inhibiting its proteasomal degradation. This leads to β-catenin accumulation and activation of the Wnt/β-catenin pathway, promoting renal interstitial fibrosis [58]. Additionally, in cisplatin-induced AKI, FAT10 modifies the tumor necrosis factor receptor (TNF-R), enhancing TNF-α-mediated apoptosis of tubular epithelial cells and exacerbating kidney injury [52]. These studies indicate that FATylation exerts a pro-injury role in the pathological process of AKI by regulating fibrosis and apoptosis, and can serve as a potential target for regulating AKI outcomes.

#### 2.3.6. Urmylation

Urmylation refers to the conjugation of ubiquitin-related modifier 1 (URM1) to substrates. URM1 is an evolutionarily conserved, ancient UBL that shares structural homology with prokaryotic sulfur carriers. The urmylation process relies on the E1-like enzyme MOCS3/UBA4 and proceeds through a thioester intermediate, but no typical E2/E3 enzymes have been identified, suggesting a unique modification mechanism [53].

In IRI-induced AKI, URM1 modifies mitochondrial oxidases (e.g., superoxide dismutase 2, SOD2) through urmylation, enhancing the stability and antioxidant activity of SOD2, reducing reactive oxygen species (ROS) production, and alleviating mitochondrial oxidative stress injury [59,60,61]. URM1-knockout mice exhibit more severe mitochondrial dysfunction and tubular injury after IRI, while exogenous URM1 overexpression can reverse this phenotype [61]. This mechanism indicates that urmylation exerts a protective role in AKI by protecting mitochondrial function and inhibiting oxidative stress, providing a new direction for antioxidant therapy in AKI.

## 3. Role of Ubiquitination and UBLs in the Pathology of AKI

### 3.1. Integrated Regulatory Networks in AKI

The ubiquitin system orchestrates a complex regulatory network that coordinates multiple pathological processes in AKI. Rather than functioning as isolated pathways, ubiquitination and UBLs create interconnected signaling cascades that link inflammation, ER stress, apoptosis, repair mechanisms, and mitochondrial dysfunction [62].

The progression from initial injury to repair or fibrosis involves sequential and overlapping ubiquitin-mediated processes: (1) Inflammation initiation through K63-linked ubiquitination of TRAF6 activating NF-κB signaling; (2) ER stress response via UFMylation and neddylation coordinating ERAD and UPR pathways; (3) Cell fate decisions through competitive SUMOylation and ubiquitination of apoptotic regulators like p53 and Bcl-2; (4) Repair activation via NEDDylation-dependent CRL activation promoting cell cycle progression; (5) Mitochondrial quality control through PINK1-Parkin ubiquitination cascades triggering mitophagy [63,64].

This integrated network explains why targeting single ubiquitin pathways often yields modest therapeutic effects, while combination approaches addressing multiple interconnected modifications show greater promise for AKI treatment.

### 3.2. Ubiquitination and Inflammation: K63 vs. K48 Chain Specificity

Inflammation represents a central pathophysiological mechanism in AKI development, with innate immune signaling pathways playing crucial roles in initiating and propagating inflammatory responses. Ubiquitination regulates multiple aspects of inflammatory signaling, particularly through modulation of the NF-κB pathway, a master regulator of pro-inflammatory cytokine production [65].

NF-κB activation requires degradation of its inhibitor, IκBα, which undergoes K48-linked polyubiquitination by the SCF^β-TrCP^ E3 ligase complex following phosphorylation by IκB kinase (IKK) [66]. Additionally, activation of IKK itself depends on K63-linked ubiquitination of upstream signaling components, including TRAF6 (TNF receptor-associated factor 6) and RIP1 (Receptor-interacting protein 1) [67]. They are summarized in Table 3.

In experimental AKI models, ischemia–reperfusion injury (IRI) induces rapid activation of NF-κB in tubular epithelial cells and infiltrating immune cells, promoting expression of pro-inflammatory cytokines such as TNF-α, IL-6, and IL-1β [70] demonstrated that inhibition of cullin-RING E3 ligases by MLN4924, a small molecule inhibitor of the NEDD8-activating enzyme, attenuated NF-κB activation and reduced inflammation in a mouse model of IRI-induced AKI.

The NLRP3 inflammasome, another critical mediator of inflammation in AKI, is also regulated by ubiquitination. Deubiquitination of NLRP3 by BRCC3 (BRCA1/BRCA2-containing complex subunit 3) is required for inflammasome activation, while K48-linked ubiquitination by FBXL2 (F-box and leucine-rich repeat protein 2) promotes its cisplatin-induced AKI degradation, increased NLRP3 inflammasome activation correlates with decreased FBXL2 expression, suggesting that dysregulated ubiquitination contributes to excessive inflammasome activity, Jefferies CA [71] research shows. Studies have shown that NF-κB activity, a key transcription factor involved in the inflammatory response, can be enhanced through the action of E3 ligases, leading to persistent activation of downstream inflammatory pathways. This persistent activation can result in cell damage and the spread of inflammation, ultimately affecting the development and progression of AKI [69]. The ubiquitination process directly impacts the stability and transport of NF-κB, as well as the expression of its downstream targets. This highlights the importance of understanding the mechanisms by which E3 ligases regulate NF-κB activity through ubiquitination in the context of inflammatory responses and AKI. Furthermore, the ubiquitin-proteasome system (UPS) has been identified as the major intracellular protein degradation system, capable of eliminating proteins involved in immune responses [72].

TLR4 is a key receptor involved in the endogenous immune response, regulating its stability and signaling through ubiquitination. In AKI, TLR4 activation not only triggers an immune response but also leads to renal inflammatory damage. TRAF6 promotes the downstream signaling of TLR4 by ubiquitinating it, thereby enhancing the inflammatory response in the kidneys [68]. ISGylation, a crucial immune regulatory mechanism, involves the attachment of ISG15 to target proteins, modulating their functions. ISGylation plays a role in AKI by regulating immune responses and antiviral reactions. Perng YC et al. [73] suggest that ISG15 may protect against AKI by influencing cellular antioxidant capacity. During AKI, kidney tissues suffer oxidative stress, elevating intracellular reactive oxygen species (ROS) levels [74]. ISG15 may help reduce ROS levels by modulating the intracellular antioxidant enzyme system, thereby alleviating oxidative stress and damage to kidney cells. However, recent evidence reveals a more complex role of ISG15 in AKI pathogenesis. Contrasting with its traditional protective role, recent studies demonstrate that ISG15 exacerbates AKI through ISGylation of NOX4, preventing its ubiquitination-mediated degradation and consequently increasing ROS production [54]. This finding aligns with recent studies showing that by promoting TGFβR1 ISGylation, ISG15 accelerates acute kidney injury and the subsequent AKI-to-CKD transition [55]. The competitive relationship between ISGylation and ubiquitination represents a critical regulatory mechanism, where ISG15 stabilizes target proteins by preventing their proteasomal degradation. These discoveries highlight the context-dependent and potentially detrimental role of ISG15 in renal pathophysiology, suggesting that therapeutic strategies targeting the ISG15/ISGylation axis warrant further investigation [75].

### 3.3. Ubiquitination and Endoplasmic Reticulum Stress (ER Stress)

In AKI, the function of ER-associated degradation (ERAD) may be impaired, resulting in enhanced endoplasmic reticulum stress and triggering tubular cell death. ERAD is a critical process for restoring ER function by regulating the unfolding of proteins and reducing the accumulation of misfolded proteins, thus alleviating ER stress [76]. Keuss MJ et al. [77] demonstrated that NEDD8 promotes proper protein folding and degradation by interacting with proteins and regulating E3 ubiquitin ligase activity, helping maintain ER function. Moreover, NEDD8 may reduce oxidative stress-induced cell death by inhibiting PARP-1 activation, thereby reducing stress responses in AKI.

### 3.4. Ubiquitination and Apoptosis

Apoptosis is a significant mechanism of renal damage in AKI. Bcl-2 and Caspase family proteins are key regulators of apoptosis. Bcl-2 family proteins are key regulators of renal tubular epithelial cell apoptosis in AKI, and their stability is precisely regulated by ubiquitination. In cisplatin-induced AKI, the E3 ligase MCL-1 modifies Bcl-2 through K48-linked ubiquitination, promoting its proteasomal degradation. This leads to a decrease in the level of the anti-apoptotic protein Bcl-2 and increased apoptosis of tubular epithelial cells [78]. In contrast, the deubiquitinating enzyme USP7 can maintain Bcl-2 stability by removing its K48-linked ubiquitination, reducing cisplatin-induced apoptosis [79]. In a cisplatin-treated AKI mouse model, USP7 knockout exacerbates Bcl-2 degradation and tubular apoptosis, while USP7 overexpression or use of a USP7 agonist (e.g., HBX 41108) significantly increases Bcl-2 levels, alleviates kidney injury, and improves renal function [79,80]. This evidence directly confirms that USP7-mediated deubiquitination of Bcl-2 is a key mechanism for inhibiting apoptosis in cisplatin-induced AKI, providing a clear target for anti-apoptotic therapy in AKI. The activity of Caspase-3 is also regulated by ubiquitination, and excessive ubiquitination of Caspase-3 may exacerbate cell death, worsening AKI pathology [81].

SUMOylation, often associated with cell survival, regulates the stability of factors such as p53 and Bcl-2 during AKI, playing a critical role in cellular responses and apoptosis. Guo C et al. [82] are exploring the regulatory factors of SUMOylation (e.g., p53, Bcl-2) and how these factors may influence apoptosis and cell responses in AKI. In addition, Cisplatin-induced SUMOylation can be reduced by antioxidants like N-acetylcysteine and dimethylurea, supporting the role of oxidative stress in SUMO activation. Therefore, studying the relationship between SUMOylation and oxidative stress may provide new insights into AKI treatment.

### 3.5. Ubiquitination and Tubular Repair

Cell cycle regulators such as Cyclins and CDKs play key roles in tubular repair. During AKI recovery, these factors regulate cell proliferation and division through ubiquitination [83]. After AKI, renal tubular epithelial cells must proliferate rapidly to restore kidney function. Cyclin D1 and CDK4 promote cell progression from the G1 to S phase, thereby driving proliferation. Several studies have demonstrated that enhancing cell cycle progression through pathways like PI3K/AKT can accelerate renal tubular epithelial cell proliferation and kidney repair, with these effects potentially mediated through Cyclin D1 and CDK4 regulation [84]. NEDD8 modification affects cell cycle progression by regulating the Cullin-RING E3 ligase complex. NEDD8 modification activates E3 ligases, influencing protein degradation and facilitating tubular cell repair and regeneration [85]. FAT10, a UBL associated with immune responses and fibrosis, plays a crucial role in AKI. Studies indicate that FAT10 modification promotes kidney fibrosis by enhancing immune cell infiltration, thereby advancing fibrosis.

### 3.6. Ubiquitination and Mitochondrial Function

Mitochondrial dysfunction represents a hallmark of AKI, with ubiquitin-mediated quality control systems determining cellular energy status and survival. The PINK1-Parkin pathway represents the best-characterized mitochondrial quality control mechanism, but recent discoveries have revealed additional layers of regulation. (1) PINK1-Parkin Canonical Pathway: Upon mitochondrial depolarization, PINK1 accumulates on the outer mitochondrial membrane and phosphorylates ubiquitin at S65, creating phospho-ubiquitin [86]. This modified ubiquitin recruits and activates Parkin through conformational changes, initiating widespread mitochondrial ubiquitination [87]. K63-linked and K27-linked ubiquitin chains on mitochondrial proteins serve as “eat-me” signals for autophagy receptors [88]. (2) Autophagy Receptor Networks: Multiple autophagy receptors recognize ubiquitinated mitochondria through distinct ubiquitin-binding domains [89]. NDP52 preferentially binds K63-linked chains and recruits LC3B through its LIR domain [90]. OPTN (Optineurin) recognizes both K63-linked and linear ubiquitin chains while simultaneously binding to LC3 and GABARAP [91]. p62/SQSTM1 serves as a general adaptor for various ubiquitin linkages but shows a preference for K63-linked chains [50]. (3) URMylation in Mitochondrial Protection: Recent studies reveal that URMylation provides an additional layer of mitochondrial protection during AKI [59]. URM1 modification of mitochondrial oxidases enhances their stability and antioxidant capacity, reducing ROS production [60]. URM1-deficient mice show increased susceptibility to cisplatin-induced AKI with enhanced mitochondrial dysfunction [61]. (4) Mitochondrial-ER Communication: The ubiquitin system coordinates mitochondrial-ER communication during stress [14]. UFMylation of ER-resident proteins influences mitochondrial calcium uptake, while mitochondrial ubiquitination signals feedback to ER stress pathways [92]. This bidirectional communication determines whether cells undergo apoptosis or activate adaptive responses (Table 4).

## 4. Deubiquitinating Enzymes (DUBs) in AKI

### 4.1. DUB-Mediated Inflammation and Cytoprotection

DUBs play critical roles in modulating inflammatory responses in AKI by regulating the stability and activity of key signaling molecules (Table 5) [93]. DUBs such as A20 and CYLD are negative regulators of the NF-κB pathway. A20 deubiquitinates IκBα, inhibiting NF-κB activation and reducing inflammation in AKI. Rogers NM et al. [94] showed that A20 removes the ubiquitin chain from IκBα, preventing its degradation, and thus maintains NF-κB in an inactive state. CYLD, a tumor suppressor, also negatively regulates NF-κB. Yajie Li et al. [95] found that a small molecule inhibitor, Subquinocin, inhibits CYLD and USP family DUBs, promoting NF-κB activation.

A20 (TNFAIP3), a protein with both DUB and E3 ligase activities, serves as a negative regulator of NF-κB signaling by removing K63-linked ubiquitin chains from signaling components such as TRAF6 and RIP1 [96].

In experimental AKI models, A20 expression is induced as a protective mechanism to limit inflammation. Ke B et al. [97] found that tubular epithelial cell-specific deletion of A20 exacerbated kidney injury and inflammation in mouse models of IRI and cisplatin-induced AKI, while A20 overexpression conferred protection. Similarly, the DUB Cylindromatosis (CYLD) negatively regulates NF-κB activation by removing K63-linked ubiquitin chains from TRAF2, TRAF6, and NF-κB essential modulator (NEMO) [98]. Thuillier R et al. [99] showed that CYLD-deficient mice exhibited enhanced NF-κB activation and increased inflammatory cytokine production following renal IRI.

Ubiquitin-specific peptidase 13 (USP13) regulates the stability of myeloid cell leukemia 1 (MCL-1) by modulating its ubiquitination status, thereby participating in the regulation of mitochondrial function. In the context of AKI, downregulated USP13 expression leads to increased MCL-1 degradation, which exacerbates mitochondrial dysfunction and promotes renal tubular epithelial cell injury. Furthermore, inhibition of USP13 further impairs the mitochondrial protective effect mediated by MCL-1, aggravating the pathological progression of AKI. These findings suggest that the USP13-MCL-1 axis represents a potential therapeutic target for AKI management [100].

### 4.2. DUBs Influence Tubular Repair

USP9X, a DUB, has been shown to enhance tubular epithelial cell proliferation by deubiquitinating target proteins, thereby aiding kidney repair [79]. Bufalieri F et al. [80] found that USP9X removes ubiquitin chains from target proteins, facilitating renal tubular cell proliferation and kidney recovery. DUBs regulate protein stability and activity, influencing proliferation, differentiation, and migration of tubular epithelial cells, thus promoting renal repair.

In a mouse model of IRI-induced AKI, tubular epithelial cell-specific deletion of USP8 impaired EGFR signaling and delayed tubular regeneration, while USP8 overexpression enhanced tubular repair [101]. Similarly, the DUB USP10 stabilizes p53 by removing ubiquitin chains added by MDM2, influencing cell cycle progression and senescence. In cisplatin-induced AKI, USP10 expression was upregulated in surviving tubular cells, correlating with increased p53 stability and cell cycle arrest, suggesting that USP10 may prevent premature proliferation of damaged cells [102].

The DUB UCH-L1regulates Wnt/β-catenin signaling by stabilizing β-catenin through deubiquitination. Following IRI, UCH-L1 expression is induced in regenerating tubular cells, correlating with increased β-catenin levels and enhanced tubular repair. Pharmacological inhibition of UCH-L1 delayed recovery from AKI, while UCH-L1 overexpression accelerated tubular regeneration, highlighting its potential as a therapeutic target for promoting kidney repair [103].

### 4.3. DUBs as Potential Therapeutic Targets

DUBs modulate cellular processes, including inflammation, apoptosis, and repair, making them valuable targets for AKI therapy. In AKI, excessive ubiquitination may cause cell damage, while DUB overactivity could hinder repair [104]. By precisely controlling DUB activity, ubiquitination can be finely tuned to balance cell survival, repair, and death.

## 5. Spatio-Temporal Dynamics of Ubiquitin Ligases/DUBs in AKI

The pathogenic role of ubiquitination and deubiquitination in AKI depends on renal compartment specificity (tubule, glomerulus, interstitium) and AKI etiology (IRI, nephrotoxins, sepsis) [55,69]. Notably, E3 ligases and DUBs show spatio-temporally specific activation, which collectively drives AKI progression [63,106].

### 5.1. Renal Compartment-Specific Dynamics

Ubiquitin-related molecular changes differ across renal compartments [68,107]. In proximal tubular epithelial cells during cisplatin-induced AKI, TRAF6 is upregulated at 24–48 h post-injury, mediating K63-linked ubiquitination of RIP1 to activate NF-κB [67,68]. Conversely, USP13 is downregulated at 12 h post-injury, enhancing MCL-1 degradation and worsening mitochondrial dysfunction [100]. In the glomerulus during sepsis-related AKI, podocyte-specific E3 ligase Cbl-b is activated within 6–12 h of sepsis onset. It promotes K48-linked ubiquitination of nephrin, a core slit diaphragm protein, thereby disrupting the glomerular filtration barrier [68,107]. In the renal interstitium during IRI-induced AKI, macrophage-derived DUB CYLD is downregulated at 48–72 h post-IRI. This reduction enhances K63-linked ubiquitination of TRAF6 in fibroblasts, initiating early interstitial fibrosis [98,99].

### 5.2. Etiology-Specific Dynamics

Activation patterns of E3 ligases and DUBs vary by AKI etiology [55,65]. In IRI-induced AKI, K63-linked ubiquitination of TRAF6 dominates the early phase (0–6 h) to trigger inflammation, while K48-linked ubiquitination of Bcl-2 becomes prominent in the late phase (48–72 h) to promote tubular apoptosis [63,67]. In cisplatin-induced AKI, K48-linked ubiquitination of NOX4 (a ROS-producing enzyme) is inhibited by ISGylation. Meanwhile, URM1-mediated urmylation modifies SOD2 at 24–72 h post-injury to alleviate oxidative stress [55,61]. In sepsis-induced AKI, linear ubiquitination of NEMO occurs in the early phase (3–6 h) in both tubular and glomerular cells, amplifying NF-κB activation and inflammation [59,64]. In the late phase (12–24 h), DUB USP18 is upregulated, which negatively regulates ISGylation and restricts type I interferon-mediated excessive inflammation to prevent further kidney injury [55,106].

The spatio-temporal specificity of E3 ligases and DUBs is critical for AKI therapy. For example, targeting TRAF6 within 6 h post-IRI reduces early inflammation, and activating USP13 at 12–24 h post-cisplatin preserves MCL-1 expression and mitochondrial function [64,100].

## 6. Ubiquitination and UBLs as Therapeutic Targets in AKI

### 6.1. Targeting E3 Ligases for Therapy

Specific inhibition of E3 ligases involved in p53 regulation, such as MDM2, represents another therapeutic strategy. The MDM2 inhibitor nutlin-3 stabilized p53 and enhanced its transcriptional activity in tubular epithelial cells, promoting cell cycle arrest and allowing for DNA repair rather than apoptosis. MLN4924, a NEDD8-activating enzyme inhibitor, blocks E3 ligase function by inhibiting NEDD8 conjugation, reducing NF-κB activation and kidney tubular damage. MLN4924 holds potential as a therapeutic strategy for AKI by modulating E3 ligase activity [101]. TRAF6 and Cbl-b, E3 ligases involved in immune responses, can be targeted to inhibit inflammation in AKI. TRAF6 promotes NF-κB activation, contributing to inflammation. Inhibition of TRAF6 may reduce kidney inflammation in AKI. These protective effects were mediated by inhibition of NF-κB signaling and stabilization of Nuclear factor erythroid 2-related factor 2 (Nrf2), a master regulator of antioxidant responses. In a mouse model of cisplatin-induced AKI, nutlin-3 treatment reduced tubular cell apoptosis and preserved kidney function [105].

### 6.2. Intervening in AKI with DUBs

DUBs influence various cellular processes, including apoptosis, repair, and oxidative responses, offering a potential therapeutic approach for AKI. In animal models, inhibitors of USP14 and activators of A20 have shown significant protective effects, highlighting DUBs as promising therapeutic targets [108]. DUBs regulate inflammation, apoptosis, and repair by modulating the ubiquitination state of key proteins.

### 6.3. UBLs as Therapeutic Targets

NEDD8 modification, through its interaction with Cullin-RING E3 ligase, promotes E3 ligase activation. Inhibition of NEDD8 conjugation by MLN4924 reduces tubular damage in AKI. ISGylation, mediated by ISG15, has immune regulatory functions, and targeting ISG15 or modulating its expression may inhibit AKI-related inflammation [109,110]. UFM1 modification plays an important role in managing ER stress and improving renal ER homeostasis. Upregulating UFM1 may help cells cope with ER stress and reduce AKI-induced damage [111,112]. FAT10 regulates fibrosis, particularly in the later stages of AKI, and targeting FAT10 could alleviate fibrosis in kidney injury.

Modulation of UBL systems, including SUMOylation, neddylation, and ISGylation, represents a promising therapeutic approach for AKI management. Targeting SUMO-specific proteases (SENPs) may provide therapeutic benefits by enhancing SUMOylation of protective transcription factors such as HIF-1α and Nrf2 [113,114].

The SENP inhibitor GN6571 increased global SUMOylation levels and protected against oxidative stress-induced tubular cell injury by enhancing Nrf2 stability and transcriptional activity [115,116]. Similarly, targeting neddylation through inhibition of the NEDD8-activating enzyme (NAE) has shown promising results in experimental AKI models [117,118].

Treatment with the NAE inhibitor MLN4924 attenuated inflammation, oxidative stress, and apoptosis in mouse models of IRI and cisplatin-induced AKI [119,120]. These protective effects were mediated by inhibition of cullin-RING ligase activity, leading to stabilization of cytoprotective proteins such as Nrf2 and IκBα (an inhibitor of NF-κB) [56,57].

Modulation of ISGylation, which is induced in response to type I interferons and plays critical roles in antiviral immune responses, may also provide therapeutic benefits in certain forms of AKI [58,121]. The ISG15-specific protease USP18 negatively regulates ISGylation and interferon signaling. In a mouse model of virus-induced AKI, USP18 deficiency exacerbated kidney injury through enhanced type I interferon responses, suggesting that USP18 augmentation may represent a potential therapeutic strategy for virus-associated AKI [106,122].

### 6.4. Multi-Pathway Combined Targeting Strategy

Given the synergistic effects of ubiquitination and UBL pathways in AKI, single-target intervention is insufficient to completely block AKI progression. For example, MLN4924 can inhibit NF-κB inflammation but cannot improve mitochondrial dysfunction; however, combined use of a URM1 agonist and MLN4924 can simultaneously alleviate inflammation and mitochondrial injury in the IRI-AKI model, significantly improving renal protective effects [48,61]. This result supports the “multi-pathway combined targeting” therapeutic strategy. Future research should further explore the synergistic mechanisms between ubiquitination/UBLs pathways to develop more effective combined treatment regimens.

### 6.5. Future Research Directions

CRISPR-Cas9 gene editing technology is increasingly used to study ubiquitination modifications. Future research could use CRISPR to modulate E3 ligases, DUBs, or UBLs to precisely investigate their roles in AKI and provide new avenues for therapeutic development [123,124,125]. PROTAC technology, which uses small molecules to target proteins for degradation, shows promise in modulating specific ubiquitination factors in AKI treatment [126,127].

**Figure 3:**
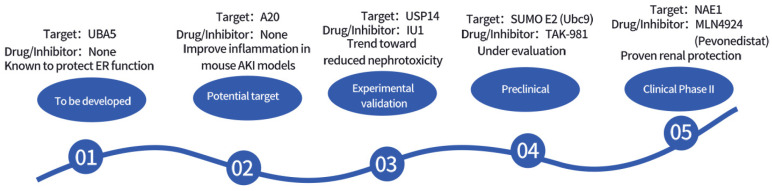
Timeline of Drug Development Targeting Ubiquitin-Related Modifications.

Figure 3 and Table 6 summarize the potential therapeutic targets and drug development prospects for ubiquitin-related modifications.

## 7. Future Research Directions and Translational Challenges

Significant progress has been made in understanding the roles of ubiquitination and UBLs in AKI, but critical mechanistic gaps and translational bottlenecks still exist. This section proposes testable research hypotheses, discusses the application potential of technologies including CRISPR-Cas9 and PROTACs, and analyzes the challenges and opportunities associated with targeting ubiquitin pathways for AKI therapy [123,126].

### 7.1. Specific Testable Research Hypotheses

To address unresolved mechanisms in AKI, two hypotheses with clear experimental validation protocols are proposed. The first hypothesis posits that targeting the USP13-MCL-1 axis can alleviate cisplatin-induced mitochondrial injury in AKI. This hypothesis is supported by evidence that USP13 stabilizes MCL-1 through deubiquitination to reduce mitochondrial apoptosis [100], and that downregulation of USP13 in cisplatin-induced AKI leads to increased MCL-1 degradation [80]. For validation, AAV-mediated tubular-specific USP13 overexpression will be used in cisplatin-induced AKI mice to detect mitochondrial function (such as reactive oxygen species levels and mitochondrial membrane potential) and renal injury markers (including serum creatinine and tubular apoptosis rate); in vitro, tubular epithelial cells treated with USP13 agonists will be used to verify the effects of USP13 activation on MCL-1 stability and mitochondrial injury [100,121].

The second hypothesis suggests that FAT10-targeted PROTACs can inhibit the transition from AKI to CKD. This hypothesis is based on findings that FAT10 stabilizes β-catenin through FATylation to promote renal fibrosis [58], and that PROTACs technology enables specific degradation of target proteins [46]. For validation, FAT10-targeted PROTACs molecules—constructed by conjugating a FAT10 antibody with an E3 ligase ligand—will be tested in a mouse model of AKI-CKD transition (e.g., the unilateral ureteral obstruction, UUO model). These experiments will assess the effects of PROTACs on FAT10 degradation, β-catenin levels, and the severity of renal fibrosis (such as the expression of α-SMA and collagen I), while also evaluating the renal targeting ability and safety of the PROTACs [21,58].

### 7.2. Technological Applications: CRISPR-Cas9 and PROTACs in AKI Research

CRISPR-Cas9 and PROTACs represent promising tools to advance AKI research, though several technical challenges need to be addressed. For CRISPR-Cas9 technology, one key application is the generation of conditional knockout mice for UBL genes—such as tubular-specific URM1 knockout mice—which can help clarify the cell-specific role of UBLs in AKI [61,123]. Another application involves upregulating protective genes like USP13 and URM1 through CRISPR activation (CRISPRa) technology, which allows researchers to evaluate the therapeutic effects of these genes on AKI [117,124]. However, this technology faces challenges, including low kidney tissue-specific delivery efficiency—for example, the transduction efficiency of AAV vectors into tubular epithelial cells is only approximately 30% to 50%—and off-target effects resulting from long-term gene editing, such as mutations in non-target genes [118].

For PROTACs technology, potential applications include the development of PROTACs targeting E3 ligases like TRAF6 and SCFβ-TrCP, which can be used to inhibit inflammation in AKI [46,66,67]. Additionally, designing dual-target PROTACs—such as molecules that degrade both FAT10 and NOX4 simultaneously—may enable synergistic inhibition of AKI-related fibrosis and oxidative stress [21,55,58]. Nevertheless, PROTACs also have technical limitations: their large molecular weight (>1000 Da) restricts penetration of the tubular epithelial cell membrane, and their short in vivo half-life (approximately 1 to 2 h) requires formulation optimization, such as nanoparticle encapsulation, to improve their pharmacokinetic properties [21].

### 7.3. Translational Challenges and Opportunities

Despite promising preclinical findings, translating ubiquitin/UBL-targeted therapies for AKI into clinical practice faces key hurdles. First, crosstalk between different pathways may trigger compensatory effects—for instance, inhibition of NF-κB signaling to reduce inflammation may inadvertently exacerbate tubular cell apoptosis [62,64]. Second, species differences limit the extrapolation of results from mouse models to humans; a notable example is the need for dose adjustments when applying drugs like MLN4924 from preclinical studies to human trials [48,118]. Third, the ubiquitin system is involved in fundamental cellular functions, so targeting this system carries off-target risks—such as inhibition of neddylation leading to immunosuppression [57].

However, several opportunities exist to advance the clinical translation of these therapies. Precision targeting can be achieved through single-cell sequencing, which helps identify pathway-specific activation in distinct renal cells—such as the tubular USP13-MCL-1 axis—and enables more targeted therapeutic interventions [124,128]. Combined treatment regimens, such as co-administration of MLN4924 with antioxidants, can enhance therapeutic efficacy by addressing multiple pathological processes simultaneously [48,61]. Additionally, developing urinary ubiquitinated proteins like FAT10 and URM1 as diagnostic or prognostic biomarkers can improve the clinical management of AKI and facilitate the evaluation of ubiquitin-targeted therapies [58,61].

## 8. Conclusions and Outlook

UBLs play a crucial role in the occurrence and progression of AKI by regulating inflammation, apoptosis, repair, and other cellular processes [128,129]. Various ubiquitination modifications (such as SUMOylation, Neddylation, and ISGylation) interact with each other to regulate the pathological progression of AKI [130]. UBLs (such as SUMO, NEDD8, and ISG15) modulate immune responses, protein degradation, and endoplasmic reticulum stress, thereby influencing the development of AKI [107,131]. Future research should focus on exploring the specific roles of these modifications and their interaction mechanisms in AKI [132,133]. By precisely regulating the signaling pathways of ubiquitination and UBLs, new therapeutic targets for AKI may be identified. Future studies could further advance the development of precision treatments for AKI through strategies such as DUB inhibitors, small molecules targeting E3 ligases, and gene editing technologies [134,135].

Although current research has provided valuable insights into the role of ubiquitination modifications in AKI, our understanding of the specific mechanisms remains incomplete [136]. Future research should focus on the roles of ubiquitination modifications in organelle function, oxidative stress, immune responses, and other aspects, while also exploring how to more precisely regulate these pathways to develop new therapeutic strategies.

## Figures and Tables

**Table 1:** Deubiquitinating enzymes and their roles in cellular processes.

Functional Category	DUB/Enzyme	Target Protein	Regulatory Mechanism	Biological Significance	References
DNA Damage Response and Protein Stability	USP1	FANCD2	Removes monoubiquitin after DNA repair	Allows cell cycle progression and maintains normal cellular functions	[32,33]
	USP4	BRCA1	Deubiquitination-mediated stabilization	Positively regulates BRCA1 stability and genomic integrity maintenance	[34]
	OTUB1	DNA repair proteins	Ubiquitin removal from repair factors	Key regulator in DNA damage repair and cellular protection	[38,39]
Protein Quality Control	BRCA1 (E3 ligase)	PERK, IRE1	Ubiquitination of UPR sensors	Controls unfolded protein response; dysfunction leads to proteostatic stress	[35]
	DUBs (general)	UPR components	Counterbalance ubiquitination	Essential for protein homeostasis maintenance in stress conditions	[36]
Signal Transduction	DUBs (general)	ABA signaling factors	Regulation of ubiquitination levels	Modulate hormone signaling strength and cellular responses to environmental stimuli	[37]
Cellular Protection	DUBs (general)	Stress response proteins	Enhanced protein stability	Promote cellular survival under various stress conditions	[40,41]

**Table 2:** Comparison Table of Functions and Characteristics of Ubiquitin-like Modifications.

Modification Type	Structural Features	Key Enzymes	Regulatory Targets	Role in AKI	References
SUMOylation	Small, structurally compact	UBC9, PIAS	p53, Bcl-2	Anti-apoptotic, protein stabilization	[42,43,44]
Neddylation	Ubiquitin-like	NAE1, UBC12	Cullin, CDKs	Initiates cell repair	[21,45,46,47,48]
ISGylation	IFN-induced	UBE1L, UBE2L6	IRF3, STATs	Antiviral, antioxidant	[37,39,49]
UFMylation	Novel UBL	UBA5, UFC1	DDRGK1, ERAD complex	Alleviates ER stress	[50]
FATylation	Affects immunity	UBA6, USE1	β-catenin, TNF-R	Promotes fibrosis and stress response	[51,52]
Urmylation	Affects tRNA modification	UBA4, URM1	Mitochondrial oxidases	Antioxidant protection	[53]

**Table 3:** Ubiquitin Chain Specificity in AKI Inflammatory Signaling.

Chain Type	Target Protein	E3 Ligase	Functional Outcome	Role in AKI	References
K63-linked	TRAF6	TRAF6 (auto)	Signal platform formation	Pro-inflammatory	[67,68]
K63-linked	RIP1	TRAF6, cIAP1/2	MAPK/NF-κB activation	Pro-inflammatory	[67]
K48-linked	IκBα	SCF^β-TrCP^	Proteasomal degradation	Pro-inflammatory	[66]
K48-linked	NOX4	Unknown	Protein degradation	Anti-inflammatory	[55]
Mixed chains	NEMO	Linear ubiquitin chain assembly complex	Enhanced NF-κB activation	Pro-inflammatory	[65,69]

**Table 4:** Mitochondrial Quality Control Components in AKI.

Component	Modification	Function	Outcome in AKI	References
PINK1	Autophosphorylation	Ubiquitin kinase activation	Mitophagy initiation	[86]
Parkin	Phospho-ubiquitin binding	E3 ligase activation	Mitochondrial ubiquitination	[87]
NDP52	K63-Ub recognition	Autophagy receptor	Mitophagy execution	[90]
OPTN	Linear/K63-Ub binding	Autophagy receptor	Enhanced clearance	[91]
URM1	Protein urmylation	Antioxidant enhancement	Cytoprotection	[59,60,61]

**Table 5:** Deubiquitinating Enzymes in Acute Kidney Injury.

DUB	Substrates	Signal Pathway	Effects	References
A20	IκBα, TRAF6, RIP1	NF-κB	Removes ubiquitin chain from IκBα, preventing degradation; maintains NF-κB in inactive state; removes K63-linked ubiquitin chains; protective mechanism to limit inflammation; A20 overexpression confers protection in AKI	[94,96,97]
CYLD	TRAF2, TRAF6, NEMO	NF-κB	Negatively regulates NF-κB activation by removing K63-linked ubiquitin chains; CYLD-deficient mice showed enhanced NF-κB activation and increased inflammatory cytokine production following renal IRI	[98,99]
USP13	MCL-1	USP13-MCL-1	Regulates MCL-1 ubiquitination to maintain its stability and participate in mitochondrial function modulation; downregulating USP13 expression in AKI leads to increased MCL-1 degradation and exacerbated mitochondrial dysfunction; USP13 inhibition further impairs mitochondrial protection and aggravates AKI pathogenesis	[100]
USP18	ISG15-conjugated proteins, NCOA4	ISG15/ISGylation, Ferroptosis	Removes ISG15 from ISGylated proteins (de-ISGylation); regulates ISG15-mediated protein stability; USP18-mediated deISGylation of NCOA4 promotes ferroptosis, potential therapeutic target in AKI	[55,75,76]
USP7	p53, MDM2, FOXO4	p53-MDM2, Oxidative stress	Stabilizes p53 by removing K48-linked ubiquitin chains; promotes cell survival under oxidative stress; dual role in regulating both p53 and MDM2 stability; critical for DNA damage response in AKI	[79,80,101]
USP22	Histones H2A/H2B, SIRT1	Epigenetic regulation, SIRT1	Removes ubiquitin from histones; regulates chromatin structure and gene expression; stabilizes SIRT1, promoting anti-inflammatory responses; protects against cisplatin-induced AKI	[102,103,104]
USP14	Proteasome substrates	Proteasome regulation	Regulates proteasome activity by removing ubiquitin chains; controls protein degradation rate; dysregulation leads to accumulation of damaged proteins and cellular stress in AKI	[101,105]
Subquinocin (inhibitor)	CYLD, USP family DUBs	NF-κB	Inhibits CYLD and USP family DUBs, promotes NF-κB activation	[95]

**Table 6:** Potential Therapeutic Targets and Drug Development Prospects for Ubiquitin-Related Modifications.

Target	Modification Type	Drug/Inhibitor	Status	Mechanism of Action	Clinical/Animal Model Studies	References
NAE1	Neddylation	MLN4924 (Pevonedistat)	Clinical Phase II	Inhibits CRL activation, reduces repair burden	Proven renal protection	[56,57,101,117,118,119,120]
A20	DUB	No available drugs	Potential target	Inhibits NF-κB overactivation	Improves inflammation in mouse AKI models	[108]
UBA5	UFMylation	None	To be developed	Involved in ER-phagy	Known to protect ER function	[111,112]
USP14	DUB	IU1	Experimental validation	Enhances protein clearance capacity	Trend toward reduced nephrotoxicity	[108]
SUMO E2 (Ubc9)	SUMOylation	TAK-981	Preclinical	Immunomodulator	Under evaluation	[113,114,115,116]

## Data Availability

No new data were created or analyzed in this study.

## Abbreviations

The following abbreviations are used in this manuscript:

AKI	Acute Kidney Injury
UPS	Ubiquitin-Proteasome System
UBLs	Ubiquitin-Like Modifiers
DUBs	Deubiquitinating Enzymes
NF-κB	Nuclear Factor-κB
NLRP3	NOD-Like Receptor Pyrin Domain-Containing Protein 3
SUMO	Small Ubiquitin-Like Modifier
NEDD8	Neural Precursor Cell-Expressed Developmentally Downregulated 8
ISG15	Interferon-Stimulated Gene 15
UFM1	Ubiquitin-Fold Modifier 1
FAT10	HLA-F-Adjacent Transcript 10
URM1	Ubiquitin-Related Modifier 1
USP	Ubiquitin-Specific Proteases
OTU	Otubain
UCH	Ubiquitin Carboxyl-Terminal Hydrolases
JAMM	JAB1/MPN/Mov34 Metalloenzymes
SAE	SUMO-Activating Enzyme
UBC9	Ubiquitin-Conjugating Enzyme 9
PIAS	Protein Inhibitor of Activated STAT
NAE	NEDD8-Activating Enzyme
UBE2M	Ubiquitin-Conjugating Enzyme E2M
UBE2F	Ubiquitin-Conjugating Enzyme E2F
RBX	RING-Box Protein
DCNL	Deneddylase-Like Protein
MDM2	Murine Double Minute 2
UbE1L	Ubiquitin-Activating Enzyme E1-Like
UbcH8	Ubiquitin-Conjugating Enzyme H8
HERC5	HECT and RLD Domain-Containing E3 Ubiquitin Protein Ligase 5
TRIM25	Tripartite Motif Containing 25
USP18	Ubiquitin-Specific Peptidase 18
UBA5	Ubiquitin-Like Modifier Activating Enzyme 5
UFC1	UFM1-Conjugating Enzyme 1
UFL1	UFM1-Specific Ligase 1
UFSP2	UFM1-Specific Protease 2
ER	Endoplasmic Reticulum
UPR	Unfolded Protein Response
TNF-α	Tumor Necrosis Factor-α
IFN-γ	Interferon-γ
UBA6	Ubiquitin-Activating Enzyme 6
USE1	Ubiquitin-Conjugating Enzyme E2S1
CKD	Chronic Kidney Disease
Wnt	Wingless-Type MMTV Integration Site Family
SOD2	Superoxide Dismutase 2
ROS	Reactive Oxygen Species
IRI	Ischemia–Reperfusion Injury
ERAD	ER-Associated Degradation
Bcl-2	B-Cell Lymphoma 2
MCL-1	Myeloid Cell Leukemia 1
Caspase	Cysteinyl Aspartate-Specific Protease
USP7	Ubiquitin-Specific Peptidase 7
CDKs	Cyclin-Dependent Kinases
PI3K/AKT	Phosphoinositide 3-Kinase/Protein Kinase B
CRLs	Cullin-RING E3 Ubiquitin Ligases
PINK1	PTEN-Induced Kinase 1
NDP52	Nuclear Dot Protein 52 kDa
LC3B	Microtubule-Associated Protein 1 Light Chain 3 Beta
OPTN	Optineurin
GABARAP	Gamma-Aminobutyric Acid Receptor-Associated Protein
p62/SQSTM1	Sequestosome 1
TRAF6	TNF Receptor-Associated Factor 6
RIP1	Receptor-Interacting Protein 1
IκBα	Inhibitor of NF-κB Alpha
IKK	IκB Kinase
NEMO	NF-κB Essential Modulator
FBXL2	F-Box and Leucine-Rich Repeat Protein 2
TLR4	Toll-Like Receptor 4
NOX4	NADPH Oxidase 4
TGFβR1	Transforming Growth Factor Beta Receptor 1
CYLD	Cylindromatosis
USP13	Ubiquitin-Specific Peptidase 13
USP22	Ubiquitin-Specific Peptidase 22
SIRT1	Sirtuin 1
USP14	Ubiquitin-Specific Peptidase 14
USP9X	Ubiquitin-Specific Peptidase 9 X-Linked
USP8	Ubiquitin-Specific Peptidase 8
EGFR	Epidermal Growth Factor Receptor
USP10	Ubiquitin-Specific Peptidase 10
UCH-L1	Ubiquitin C-Terminal Hydrolase L1
MLN4924	Pevonedistat
Cbl-b	Casitas B-Lineage Lymphoma Proto-Oncogene B
nephrin	Nephrin
SCFβ-TrCP	Skp1-Cullin-F-box Beta-Transducin Repeat-Containing Protein
Nrf2	Nuclear Factor Erythroid 2-Related Factor 2
SENPs	SUMO-Specific Proteases
HIF-1α	Hypoxia-Inducible Factor 1 Alpha
CRISPR-Cas9	Clustered Regularly Interspaced Short Palindromic Repeats-CRISPR-Associated Protein 9
PROTACs	Proteolysis-Targeting Chimeras
AAV	Adeno-Associated Virus
UUO	Unilateral Ureteral Obstruction
α-SMA	Alpha-Smooth Muscle Actin
FOXO4	Forkhead Box O4
